# Planning the Autumn Campaign

**Published:** 1921-08-20

**Authors:** 


### The Hospital

The Workers' Newspaper of Administrative Medicine and Institutional Life. Administration. National Insurance and Health.

No. 1837, Vol. LXX. SATURDAY, AUGUST 20, 1921. PRICE TWOPENCE

### PLANNING THE AUTUMN CAMPAIGN.

August and September are the months in which hospital managers generally devise plans for the appeal which is usually issued after the end of the summer holidays. The harvest of voluntary finance is reaped, as a rule, in the autumn, and experience seems to show that money for charity flows more freely between Michaelmas and Christmas than at other times of the year. This being so, we trust that all hospital managers who are planning an autumn campaign will remember that this year the guiding principles must be different from those which they have hitherto pursued. In the general shake-up which occurred in the voluntary system since the war, and culminated in the report of the Cave Committee and the formation of the Voluntary Hospitals Commission, many hospitals have realised the change. But as reports reach us from different parts of the country concerning the financial plans which the hospitals are laying for the autumn, it is plain that all do not recognise the new principles—namely, that appeals are now made for regular income rather than for donations for a special purpose, and that the appeal of one hospital will have to be co-ordinated with another under the pressure of the new local hospital committees. The circular to London hospitals issued by the King's Fund which we epitomise on the next page may well be studied with profit in this connection by all hospitals irrespective of situation.

There is a general and healthy recognition that each hospital's share in the forthcoming Government grant will depend upon its ability to raise a similar sum, and that special attempts to raise money should be prepared well in advance of the time when the distribution will occur. But even these facts are sometimes overlooked. Nor are the appeals in prospect always planned in regard to the requirements of the local hospital committees now in process of formation. It will be remembered that the functions of the local committees are "to marshal appeals," to "organise systematic contributions by wage-earners in areas where no such system at present exists," and "to stimulate and direct local effort." There are many places where no co-ordination at present exists, and a diminishing number, especially in the provinces, where there is "no system" of regular contributions. Consequently, the business of perhaps most of the local committees will be to insist upon co-operation and to stimulate contribution schemes. All plans for the autumn should carefully bear this prospect in view. Indeed, no such plan is well-judged until its authors have considered how far it is adapted to fit in with the requirements of the new local committees. The hospital in the strongest position in regard to its local committee will be not only that most in need, but that which can show that it has made the greatest efforts to organise new sources of revenue and to relate its resources and needs to those of the other hospitals in its area.

Whatever the local committees at first may lack, and, like all new bodies, they will certainly have their difficulties, they have at least, if not exactly the power of the purse, at least the key to it. Recommendations for a grant must come through them. But since they will be concerned, not with one hospital, but all within their area, is it not clear that autumn plans should not be drafted without consideration of the plans of neighbouring hospitals and the situation of the county in this respect? Since it will be the duty of the local committees, in the words of the Cave Committee's Report, "to marshal appeals for funds, to act as a clearing-house for patients, to promote the grading and the co-operation of the hospitals in their area, and . . . (by their control of grants or funds entrusted to them, and otherwise) to bring pressure upon hospitals when required," the hospitals should prepare their plans in the light of these activities.

The local committees are officially appreciated because they are "independent of the hospitals," and this independence is held to be necessary, because the hospitals are too much isolated at present. Since the "normal unit" is to be the county or county borough, the hospitals within this unit would do well to begin to think in terms of that area, and, so far as may be, to consider joint plans, since any isolated proposals will run the risk of coming to nought if they cannot be merged into the common scheme to be encouraged by the new local committees. In the plans which reach us we still hear too much of independent needs and plans and too little of the co-operation which the local committees have been appointed to enforce.

